# Nitrate Uptake and Transport Properties of Two Grapevine Rootstocks With Varying Vigor

**DOI:** 10.3389/fpls.2020.608813

**Published:** 2021-01-18

**Authors:** Landry Rossdeutsch, R. Paul Schreiner, Patricia A. Skinkis, Laurent Deluc

**Affiliations:** ^1^Department of Horticulture, Oregon Wine Research Institute, Oregon State University, Corvallis, OR, United States; ^2^USDA-ARS Horticulture Crops Research Unit, Corvallis, OR, United States; ^3^Oregon Wine Research Institute, Corvallis, OR, United States

**Keywords:** grapevine vigor, rootstock, nitrate uptake, N transport, carbohydrate status, transpiration

## Abstract

In viticulture, rootstocks are essential to cope with edaphic constraints. They can also be used to modulate scion growth and development to help improve berry yield and quality. The rootstock contribution to scion growth is not fully understood. Since nitrogen (N) is a significant driver of grapevine growth, rootstock properties associated with N uptake and transport may play a key role in the growth potential of grafted grapevines. We evaluated N uptake and transport in a potted system using two grapevines rootstocks [Riparia Gloire (RG) and 1103 Paulsen (1103P)] grafted to Pinot noir (Pommard clone) scion. Combining results of nitrate induction and steady-state experiments at two N availability levels, we observed different responses in the uptake and utilization of N between the two rootstocks. The low vigor rootstock (RG) exhibited greater nitrate uptake capacity and nitrate assimilation in roots after nitrate resupply than the more vigorous 1103P rootstock. This behavior may be attributed to a greater root carbohydrate status observed in RG for both experiments. However, 1103P demonstrated a higher N translocation rate to shoots regardless of N availability. These distinct rootstock behaviors resulted in significant differences in biomass allocation between roots and shoots under N-limited conditions, although the overall vine biomass was not different. Under sufficient N supply, differences between rootstocks decreased but 1103P stored more N in roots, which may benefit growth in subsequent growing seasons. Overall, greater transpiration of vines grafted to 1103P rootstock causing higher N translocation to shoots could partially explain its known growth-promoting effect to scions under low and high N availability, whereas the low vigor typically conferred to scions by RG may result from the combination of lower N translocation to shoots and a greater allocation of biomass toward roots when N is low.

## Introduction

In response to the outbreak of Grape phylloxera (*Daktulosphaira vitifoliae*) in European *Vitis vinifera* vineyards during the 19th century, grafting vines to North American *Vitis* species and their hybrids as rootstocks became commonplace worldwide. While the primary goal was to prevent vine decline due to phylloxera, there became apparent other benefits imparted to scions by rootstocks to thrive under biotic and abiotic constraints. Additionally, rootstocks were found to influence scion development and yield, which could be used to modulate cultivar traits and improve grape and wine quality (Jones et al., 2009; Ollat et al., 2016; Zhang et al., 2016).

Rootstocks influence whole plant development since they serve as the vines’ root system. Genetic variability among rootstocks can result in differences in water and nutrient uptake and transport, and the regulation of hormones and other long-distance signal molecules that impact scion growth and development (Albacete et al., 2015; Zhang et al., 2016; Gautier et al., 2019; Ibacache et al., 2020). Apart from water, nitrogen (N) is the most limiting abiotic factor that affects plant growth. In grapevine, N accumulation in shoots is positively correlated to scion growth, and is influenced by the rootstock genotype (Nikolaou et al., 2000; Zerihun and Treeby, 2002; Ibacache et al., 2020). Moreover, the impact of rootstocks on scion growth is stronger under N limitation (Lecourt et al., 2015), indicating that N uptake and transport to the shoot may explain differences observed among rootstock genotypes.

Nitrate is the main source of N taken-up by plants from soil and acts as a signal molecule to regulate its own uptake, transport, and assimilation (Crawford and Glass, 1998). Plants actively take up nitrate through a proton/nitrate-coupled mechanism mainly mediated by Low and High Affinity Transport Systems (LATS and HATS, respectively) (Lejay and Gojon, 2018; Wang Y.-Y. et al., 2018). In grapevines and other plants, HATS and LATS are rapidly (<24 h) induced in response to increasing nitrate availability (Pii et al., 2014; Tomasi et al., 2015), and the genotype of the root system modulates (i) the kinetics of HATS (Kulmann et al., 2020) and (ii) the intensity of its induction response (Tomasi et al., 2015). However, no information is available on the variability of LATS among grapevine rootstocks. In addition, some inconsistencies in the properties of HATS (Yang et al., 2007; Tomasi et al., 2015; Kulmann et al., 2020) make it difficult to draw conclusions about the relationship between nitrate uptake capacity of a given rootstock and the biomass growth conferred to scions. While the specific location of nitrate uptake among different parts of the overall root system is unclear, the youngest roots that still have an intact cortex and account for most of the overall length of roots within a given root system are thought to contribute most to ion uptake (Kozlowski and Pallardy, 1997). A study in various trees found that younger primary roots had the highest net flux of NH_4_^+^ and NO_3_^–^ even though corky and woody root zones showed some net N uptake (Hawkins et al., 2014). In grapevines, the rate of nitrate uptake was two to three times higher in 1 day-old root tips than root tips that were 10–20 days-old (Volder et al., 2005).

Gene expression analyses of *Arabidopsis thaliana* for the closest homologs to major nitrate transporters and N-signaling genes were performed in grapevine in response to nitrate resupply (Pii et al., 2014; Tomasi et al., 2015; Cochetel et al., 2017, 2019). Expression of *VitviNRT2.4A* and *VitviNRT3* homologs in SO4 rootstock (Pii et al., 2014) or of *VitviNAXT*, a putative nitrate efflux transporter, in K5BB rootstocks (Tomasi et al., 2015), seem to align with the induction response of HATS. Using genome-wide expression analyses, Cochetel et al. (2017) reported on differential responses between two rootstocks 3 and 24 h after nitrate resupply. In that experiment, nitrate accumulation in roots was associated with variations between rootstocks in the expression of genes involved in N metabolism and transport. Surprisingly, the rootstock with the lowest vigor showed the greatest nitrate accumulation and the greatest induction of genes involved in nitrate transport and assimilation (Cochetel et al., 2017). These results suggest that net nitrate uptake may be uncoupled from scion growth promotional effects of the rootstock.

Root-to-shoot transport of nitrate participates to the regulation of N shoot status and its influence on scion growth. Rootstock contribution to nitrate xylem loading and transpiration flow may aid to the differences in N shoot status observed in scions (Zhang et al., 2016; Ibacache et al., 2020). Xylem loading flux is regulated by the balance between nitrate loading and unloading in root pericycle cells. The movement of nitrate from cell to cell could either be passive, through anion channels like X-QUAC proteins (Köhler et al., 2002) or active, through members of NRT1/NPFs proteins family (Lin et al., 2008; Li et al., 2010; Léran et al., 2013; Taochy et al., 2015). In grapevine, differences in gene expression of nitrate transporters including one associated with xylem loading (VitviNAXT1) was observed between rootstocks under normal growing conditions (Henderson et al., 2014). Varying N concentrations in xylem exudate observed in ten rootstocks grafted to Thomson Seedless were found also (Nikolaou et al., 2000). Altogether, this may suggest a different ability of rootstocks to regulate the net xylem loading of nitrate in grapevine.

Plant transpiration also contributes to nitrate root-to-shoot transport directly by regulating xylem sap flow and indirectly by increasing nitrate availability through the control of the mass flow movement in soil (Cramer et al., 2009). The tight relationship between water and nitrate transport in plants may depend on the regulation of aquaporin activity and root hydraulic conductivity by nitrate availability (Tyerman et al., 2017). In grapevine, the rootstock contribution to scion transpiration has been extensively studied in the context of water deficit and scion growth response (Ollat et al., 2016; Zhang et al., 2016). The strong correlation between scion growth, transpiration, and rootstock hydraulic conductance suggests that rootstocks can modulate the translocation of N toward shoots by controlling the plant water flow (Gambetta et al., 2012; Zhang et al., 2016). Likewise, the graft union could influence translocation of N to the shoot and the transport of N-reserves to roots. The extent of healing at the graft union can alter local xylem anatomy locally (Goncalves et al., 2007). As a consequence, hydraulic properties of the whole plant could be modified with an impact on the movement of water, hormones, and other nutrients like NO_3_^–^ and NH_4_^+^ (Atkinson et al., 2003). Scion development is often altered in grafted plants with significant effects on transpiration and vigor (Edwards et al., 2014). Conversely, root growth can be impacted via poor phloem connections at the graft union that limits shoot-to-root transport of assimilates (Bieleski, 2000; Olmstead et al., 2006). Finally, as nitrate reduction mainly occurs in grapevine leaves (Zerihun and Treeby, 2002), the rootstock contribution to N translocation toward shoots may modulate the rate of N assimilation and thus the plant N status.

Nitrate reduction and assimilation are essential regulators of nitrate uptake and transport (Dechorgnat et al., 2011; Tegeder and Masclaux-Daubresse, 2018). The dependence of carbon (C) resource(s) for amino acids and proteins synthesis requires a fine regulation of the C/N balance in plants. C and N assimilates can stimulate or repress nitrate uptake and transport, respectively. Furthermore, C/N balance regulates C and N assimilation steps, which in turn affects the source-sink relationship between the root and shoot systems, and controls growth and biomass allocation (Nunes-Nesi et al., 2010; Wang and Ruan, 2016). In grapevine, biomass allocation responds to the internal C/N balance (Grechi et al., 2007) that could be modulated by rootstocks through their influence on C and N assimilation in scion leaves (Zerihun and Treeby, 2002; Zhang et al., 2016). Although scion genotype seems to play a major role on the N uptake regulation (Tomasi et al., 2015) and biomass partitioning (Tandonnet et al., 2010), the rootstock influence on C/N balance was never properly assessed in the context of shoot growth response to rootstocks under N-limited supply.

The main goal of the current study was to further understand the mechanisms of rootstocks on the N-driven scion growth response. Our objectives were to measure root nitrate uptake properties when nitrate was resupplied to plants after N-starvation in two rootstocks [Riparia Gloire (RG) and 1103 Paulsen (1103P)] known to differ in vigor conferred to the scion. We also wanted to determine if the rootstocks would influence the rate of nitrate translocation to the scion under two levels of N supply. Additionally, we evaluated the rootstock influence on C/N balance in roots and leaves, and their relationship with plant growth and biomass partitioning. Our results confirmed higher nitrate uptake capacity and N-signaling responses of RG root tips after N-resupply. However, under steady state nitrate supply, nitrate concentration in xylem sap and transpiration flow contributed to a higher delivery of nitrate to shoots when vines were grafted to 1103P. Rootstocks did not alter total plant biomass but changed partitioning under N-limited conditions, resulting in higher shoot growth performance in vines grafted to 1103P. The rootstock genotype seems to control C and N metabolites partitioning between leaves and roots that could triggers the observed variability in nitrate uptake, sensing and biomass partitioning. Overall, this study identified that nitrate translocation to shoots rather than nitrate uptake capacity of rootstocks could modulate N-driven vigor of the scion under N-limited conditions.

## Materials and Methods

### Grapevine Material

One-year-old Riparia Gloire (RG) and 1103 Paulsen (1103P) rootstocks grafted to *Vitis vinifera* “Pinot noir” clone FPS04 (91 Pommard) scion were re-potted in 6 L pots filled with sand and grown in a semi-controlled greenhouse with a dark/light regime of 8/16 h, a temperature (day/night) of 25/20°C and a minimum light intensity of 400 μmol m^–2^s^–1^ of photosynthetically active radiation, supplied by 1000 W phosphor coated metal-halide lamps (General Electric, United States). Two weeks after budbreak, plants were pruned to maintain a single shoot, and emerging lateral shoots were removed during experiments.

### Nitrate Uptake Induction and Kinetic Experiments

Eight plants per scion/rootstock combination were watered daily for 8 weeks with 0.5 N solution (N-NO_3_; Table 1) and then subjected to nitrate depletion with 0 N solution for 10 days. One hour after daylight, plants were divided into two groups each receiving either 2 L of 0 N solution supplemented with 0.5 mM of Ca(NO_3_)_2_ (induced plants) or with 0.5 mM of CaSO_4_ (non-induced plants). Roots tips (≈2 cm) were collected at six time points (0, 2, 4, 8, 12, and 24 h) through precut windows (≈40 cm^2^) on the sides of the pots and immediately immersed in root harvest solution (Table 1). Within 5 min of sampling, young roots were divided into three sub-samples within 5 min: one sample (≈300 mg) was snap frozen in liquid nitrogen and stored at −80°C until mRNA extraction; the two other samples (≈200 mg) were immediately subjected to ^15^N uptake assays at 0.2 and at 1 mM K^15^NO_3_ availability. One extra young root sample (≈200 mg) was collected at 0 and 24 h for analysis of metabolites. Labeled ^15^N uptake kinetic parameters were determined in a separate experiment for the time point 8 h using identical plants and growing conditions as described above. The only difference was that ^15^N uptake assays were carried out at the following concentration: 0.05; 0.1; 0.2; 0.3; 0.4; 0.5; 0.75; 1; 2; 5; 10 mM of K^15^NO_3_ availability.

**TABLE 1:** Macro (mM) and micronutrient (μM) composition for the different experiments conducted in the study.

Nutrients	0.5 N Solution	0 N Solution	Root harvest solution	LN	HN
Ca(NO3)_2_	0.25	–	–	–	–
CaSO_4_	–			0.3	0.6
CaCl_2_	–	–	–	1.39	1.27
KNO_3_	–	–	–	0.8	2.45
KH_2_PO_4_	0.5			–	0.57
K_2_HPO_4_	–	–	–	0.61	–
K_2_SO_4_	0.7			0.4	–
KCl	0.1			–	–
MgSO_4_	0.5			0.69	0.69
Micronutrients	1X	1X	1X	1X	1X
MES (pH 5.8)			1		

**Micronutrients**	**Final concentration (μM)**

NaFe-EDTA	100
H_3_BO_3_	10
MnSO_4_	0.5
ZnSO_4_	0.5
CuSO_4_	0.2
Na_2_MoO_4_	0.07

*pH of 0.5 N and 0 N solutions were adjusted to 5.8.*

### ^15^N Uptake Assays and Analysis

Assays were performed by exposing the young roots (≈200 mg) for 5 min to K^15^NO_3_ (99% ^15^N) solutions in 1 mM MES buffer (pH = 5.8) and supplemented with 0.5 mM CaSO_4_. Roots were then rinsed 1 min with 0.5 mM CaSO_4_, before snap-freezing the samples and storage at −80°C. Following the grinding using a mixer mill (MM 400, Retsch GmbH, Haan, Germany), root samples were freeze dried (LABCONCO Lyph Lock 6). The amount of ^15^N in the freeze-dried roots was analyzed at the Oregon State University, Stable Isotope Research Unit, via an elemental analyzer interfaced with a mass spectrometer (Sercon GSL, PDZ Europa 20/20; Sercon, Cheshire, United Kingdom).

### Steady-State Experiment

Eight plants per scion/rootstock combination were grown for 12 weeks under low (0.8 mM; LN, Table 1) and high (2.4 mM; HN, Table 1) nitrogen solutions (Lecourt et al., 2015). Stem length was measured weekly and the dry mass of roots, trunk, stem and leaves was recorded after drying the tissues at 60°C for 72 h at harvest. One day before harvest, two liters of LN or HN solution containing K^15^NO_3_ (50% ^15^N) were applied 1 h after daylight to evaluate the effect of nitrogen supply on nitrate root-to-shoot transport. At time 0, 4, 8, 12, and 24 h, one leaf per plant was selected and sequentially subjected to a measure of transpiration, xylem sap collection, surface area measurement, and finally collected for metabolite analyses. Selected leaves were fully expanded, fully exposed to light and located at similar internodes between plants, which is in the upper part of the plant (2/3 of plant height). Young root samples (≈400 mg each) were also collected at the same time points through precut windows (≈40 cm^2^) on the side of the pots and rinsed 1 min in 0.5 mM CaSO_4_ to remove surface contaminants. Leaves, roots, and xylem sap samples were snap frozen upon collection. Leaf transpiration was measured using a Li-6400XT photosynthesis system (Li-Cor, Inc., Lincoln, NE, United States) according to manufacturer’s instructions, and setup for ambient conditions in terms of temperature and relative humidity. The leaf xylem sap was collected using a Scholander pressure chamber (PMS Instruments, Alban, OR, United States). An overpressure of 0.5 MPa was applied after reaching the leaf water potential, the first drop was removed, and xylem sap was collected with a pipette within 5 min. Homogenized xylem sap sample (5 μL) and 50 μg of N (K^14^NO_3_) were applied onto an acid washed Whatman paper (10 mm^2^) and dried overnight at room temperature. ^15^N was determined on this subsample as previously described. Root and leaves samples collected at time 0 were used for metabolites quantification.

### Gene Expression Analysis

Total RNA was extracted from 100 mg fresh powder of young root tissue according to Reid et al. (2006) with slight modifications. Genomic DNA contamination was removed using the Turbo DNA-free kit (Life technologies). Reverse transcription was performed using M-MuLV Reverse Transcriptase (New England Biolabs) using oligo dT primers with 2 μg of total RNAs. Transcript abundance was quantified on a Bio-Rad CFX96 machine using SsoAdvanced^TM^ Universal SYBR^®^ Green Supermix (Bio-Rad). PCR efficiency for each primer pair was calculated using LinRegPCR (Ruijter et al., 2009). Relative genes’ expression was calculated using the 2^ΔΔ*Ct*^ method (Livak and Schmittgen, 2001) with the VitviACT7 ortholog as reference gene for normalization. Studied genes and primers used are presented in Table 2. Each PCR product were amplified first from cDNA roots libraries, cloned into a pGEM-T Easy vector (Promega, United States), and the sequence of the amplicons were confirmed by Sanger Sequencing (Center for Genome Research and Biocomputing, CGRB – OSU).

**TABLE 2:** List of analyzed genes.

Gene Identification	Gene Annotation	Name in the manuscript	Sequence of forward primer (5′− > 3′)	Sequence of reverse primer (5′− > 3′)
Vitvi04g01613	Actin 7	VitviACT7	GTGTGGAGGGATTTATCTGTAATG	CAATCACTCTCCTGCTACAAAC
Vitvi02g00529	Nitrate transporter NRT1.1	VitviNRT1.1	CAGTTCTTATTGGTGGGTGC	CCCTAATGAAAGTGTGCTCAAA
Vitvi02g00683	Proton-dependent oligopeptide transport (POT) family protein	VitviNRT1.5	AGACACCTGATGGGCTAAAGAG	TGCTGTCAAGGTGGCTAGGA
Vitvi06g01301	High-affinity nitrate transporter 2.4A	VitviNRT2.4A	CCTCCCACCTTCAAAGGA	CATGGGATGGTGTAGAGTTGG
Vitvi08g01004	High-affinity nitrate transporter 2.4B	VitviNRT2.4B	AGGTGGGAACTTCGGATC	TCTTTGGAGGGCGGTAGC
Vitvi17g00936	Nitrate transporter 3.1	VitviNRT3	TGGGTGGAGAAGAGAAAGAG	CAAGTAAACCACAGATACATCCTC
Vitvi06g00354/Vitvi06g01667	Nitrate excretion transporter1	VitviNAXT	TTCCGGGACAAGTTTCGCTC	ACCGCAGTGCTCAGATAGAATG
Vitvi18g00326	Nitrate reductase	VitviNR	TCTTTGCTGGTGTTCTGAATG	ACGCTAGGAGAGAAGAAGAAC
Vitvi05g00403	Glutamine synthetase	VitviGS2	AGGGACTCAGAGAACAATCACC	CCAGGAAGACACAACCACCAC
Vitvi17g00975	BT1 (BTB and TAZ domain protein 1)	VitviBTB	CATGTCTTCCTTGTCCTTGC	AAACAACCAGTAACCCAACG
Vitvi10g01887	CBL- interacting protein kinase 23	VitviCIPK23	GGCCCCTTCGCTATACATGG	GATGCCACACCAGACCCATT
Vitvi04g00464	BZIP protein HY5 (HY5)	VitviHY5	GAGGAGATCAGAAGAGTGCCAGAG	TTTGTCAGCCGGGCTTCTTC

### Metabolite Analyses

Approximately 30 mg of freeze-dried tissue (LABCONCO Lyph Lock 6) was extracted three times in a prechilled methanol/chloroform/water extraction solution (2.5/1/1 – v/v/v). The mixture was vortexed for 1 min and centrifuged for 5 min at 20,000 *g* at 4°C. Supernatants of the three extracts were pooled and mixed with equal volumes of chloroform and HPLC-grade water, vortexed and then centrifuged at 20,000 *g* for 5 min. The aqueous phase was collected for colorimetric assays of nitrate (Cataldo et al., 1975), Free Amino Acids (FAA) (Sun et al., 2006) and Total Soluble Carbohydrate (TSC) (Edwards et al., 2011). The remaining pellet was dried under vacuum overnight, and 5 mg was sampled for insoluble starch analysis (Edwards et al., 2011). TSC, starch and FAA concentrations were expressed as fructose, glucose and leucine equivalents.

### Statistics and Multivariate Analyses

All statistical analyses were performed using the R software (R Core Team, 2017). In the induction experiment, paired *t*-tests and Welch paired-tests were performed for each rootstock, using t0 as control, to evaluate the induction of nitrate uptake rate and metabolite changes. Comparison between two means using student *t*-test, Welch *t*-test or Wilcoxon test depending on normality or homoscedasticity of the data were conducted to validate the nitrate kinetic induction at each nitrate concentration, and to evaluate rootstock effects on nitrate uptake rate. An Analysis of Covariance was performed to compare nitrate uptake rate between rootstocks, and in response to nitrate resupply. Rootstock, time, and treatment effects on gene expression were evaluated by one-way Analysis of Variance (ANOVA). In the steady-state experiment, a two-way ANOVA was performed to compare ^15^N flow differences between rootstock and treatment, and to compare biomass allocation and metabolite concentration changes between rootstock and time. For this dataset, the ANOVA was followed by a Tukey’s multiple test from log-transformed data when assumptions were not met from the raw data.

## Results

### Nitrate Uptake Induction Response

**FIGURE 1:**
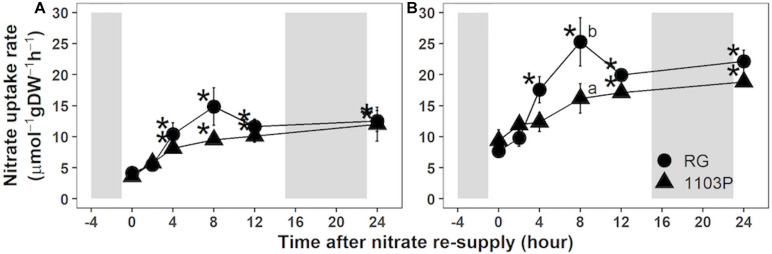
Nitrate uptake rate in roots of Riparia Gloire (circle) and 1103 Paulsen (triangle) rootstocks grafted to Pinot Noir scion (mean ± se; *n* = 4). Plants were grown 10 days without N, and then resupplied with 0.5 mM Ca(NO_3_)_2_. At the indicated times, young roots samples were transferred to 0.2 mM **(A)** or 1 mM **(B)** K^15^NO_3_ solution for 5 min (as detail in “Materials and Methods”). For each rootstock, asterisks show significant difference to the untreated sample (Time = 0 h; Paired *t*-test; *P* < 0.05) whereas letters refer to significant difference between rootstocks at each time point (Wilcoxon test; *P* < 0.05). Shaded areas represent dark period.

Nitrate uptake rate of fine roots of both rootstocks increased upon nitrate resupply (Figure 1). At the lowest concentration (0.2 mM), rootstocks increased their nitrate uptake capacity after 4 h of nitrate exposure and maintained a high rate for up to 24 h (Figure 1A). At 1 mM nitrate, RG experienced an abrupt increase 4 h after the resupply with a maximum of uptake at 8 h, followed by a decrease 12 h after the treatment (Figure 1B). A steady state uptake was then observed until the end of the experiment. For 1103P, the 1 mM resupply progressively increased nitrate uptake capacity up to 12 h before plateauing at 24 h (Figure 1B). The maximum difference in nitrate uptake capacity between rootstocks was observed at 8 h, and this time point was chosen to compare their nitrate uptake kinetics.

### Nitrate Uptake Kinetic

**FIGURE 2:**
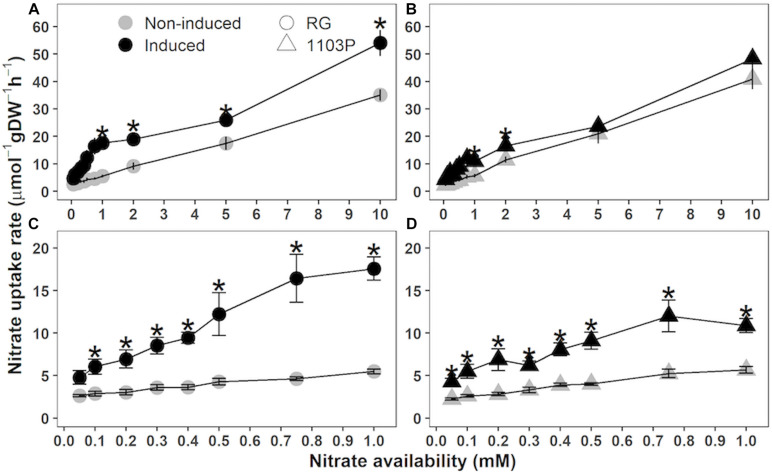
Nitrate uptake kinetics in roots of Riparia Gloire (**A,C**, circle) and 1103 Paulsen (**B,D**, triangle) rootstocks grafted to Pinot Noir scion (mean ± se; *n* = 4). Plants were grown 10 days without N, and then resupplied with 0.5 mM Ca(NO_3_)_2_ (Induced, black) or with 0.5 mM CaSO_4_ (non-induced, gray). After 8 h, young roots samples were transferred to a solution of K^15^NO_3_ in 0.05–10 mM concentration range. Asterisks show significant difference between induced and non-induced samples (*t*-test, *P* < 0.05).

For both rootstocks, nitrate uptake measurements with a gradient of nitrate availability ranging from 0.05 to 10 mM, revealed the presence of the two inducible systems for nitrate transport [Low Affinity Transport (LATS, Figures 2A,B) and High Affinity Transport Systems (HATS, Figures 2C,D)]. At high concentration (>1 mM), nitrate uptake rate increased in a nearly linear fashion to nitrate concentration with no sign of saturation up to 10 mM (Figures 2A,B). The 8-h exposure to 1 mM of nitrate significantly increased the capacity of LATS in both rootstocks (ANCOVA; RG, *p* < 0.001; 1103P, *p* = 0.003) although the magnitude of response was larger in RG compared to 1103P. The comparison between rootstocks revealed a greater LATS capacity for non-induced 1103P in comparison with non-induced RG (ANCOVA; *p* = 0.041). The opposite trend is observed in nitrate induced plants of each rootstock (ANCOVA; *p* = 0.025). At low nitrate availability (<1 mM), nitrate uptake rate followed a biphasic Michaelis-Menten kinetic pattern in both rootstocks (Figures 2C,D) with a separation of the kinetic phases occurring at 0.3–0.4 mM. For all the concentrations tested and for both rootstocks, exposure to nitrate induced an increase in nitrate uptake capacity. Kinetics parameters (Vmax and Km) could be calculated separately for each rootstock using either Michaelis-Menten or Lineweaver–Burk models, but both rootstocks could not satisfy the regression statistics on the same enzyme kinetics model. However, a general observation suggests that the two rootstocks share a common saturable component value (Vmax) for the first kinetic phase (0.05–0.4 mM), but the Vmax appeared to be higher for RG during the second kinetic phase (0.5–1 mM). In support of this observation, the nitrate uptake rate measured at 1 mM availability for the induced vines was significantly higher for RG than 1103P (*t*-test, *p* = 0.005).

### Transcript Abundance of Nitrogen Transport, Assimilation, and Signaling Following Nitrate Resupply

**FIGURE 3:**
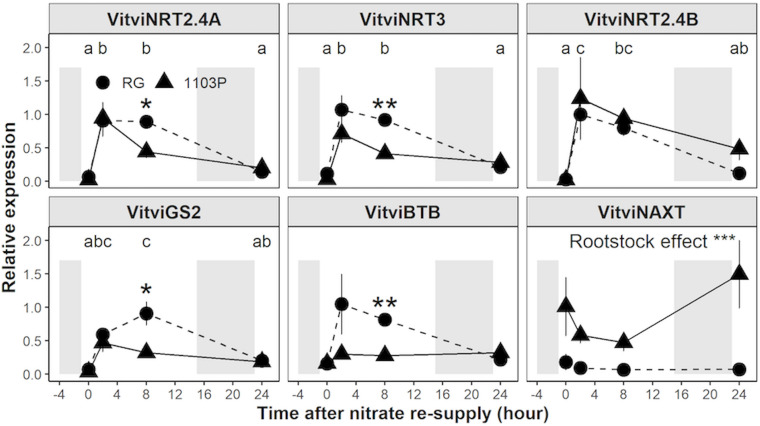
Relative gene expression of *VitviNRT2.4A, VitviNRT3, VitviNRT2.4B, VitviGS2, VitviBTB*, and *VitviNAXT* in roots tips (mean ± se; *n* = 3) of RG (circle) and 1103P (triangle) in response to nitrate resupply. Indicated time correspond to exposure duration to 0.5 mM Ca(NO_3_)_2_ after a 10 days period of N-starvation; shaded areas represent dark period. Letters refers to a significant difference between time point considering the two rootstocks (ANOVA, *P* < 0.05). Asterisks show significant difference between rootstocks at each time point (*t*-test, *P < 0.05, **P < 0.01). Overall rootstock differences are presented (ANOVA, ***P < 0.001).

Using Real-Time PCR, we monitored in both rootstocks the expression of the closest ortholog genes involved in nitrate uptake and assimilation (Figure 3 and Supplementary Figure 1). Expression profiles for *VitviNRT1.1*, *VitviNRT2.4A*, *VitviNRT2.4B*, *VitviNRT3*, *VitviNR*, and *VitviGS2* showed a strong induction 2 h after nitrate resupply followed by a down-regulation upon 24 h. Significant differences between rootstocks for the expression of *VitviNRT2.4A*, *VitviNRT3*, and *VitviGS2* appeared at 8 h with a higher expression for RG than for 1103P (Figure 3). Relative expressions of *VitviNAXT* and *VitviBTB*, involved in nitrate efflux (Segonzac et al., 2007) and nitrate signaling (Araus et al., 2016), respectively, were significantly affected depending on the rootstock genotype with a higher expression level for 1103P and RG, respectively. Rootstock differences for *VitviBTB* expression was observed mainly for the time 2 and 8 h while the level of expression was identical between rootstocks at 0 and 24 h. Expression of *VitviHY5* and *VitviCIPK23*, whose orthologs in other plants are regarded as major regulators of the nitrogen signaling, were not affected by rootstocks or by nitrate resupply (Supplementary Figure 1).

### Metabolite Dynamics in Root After Nitrate Resupply

**FIGURE 4:**
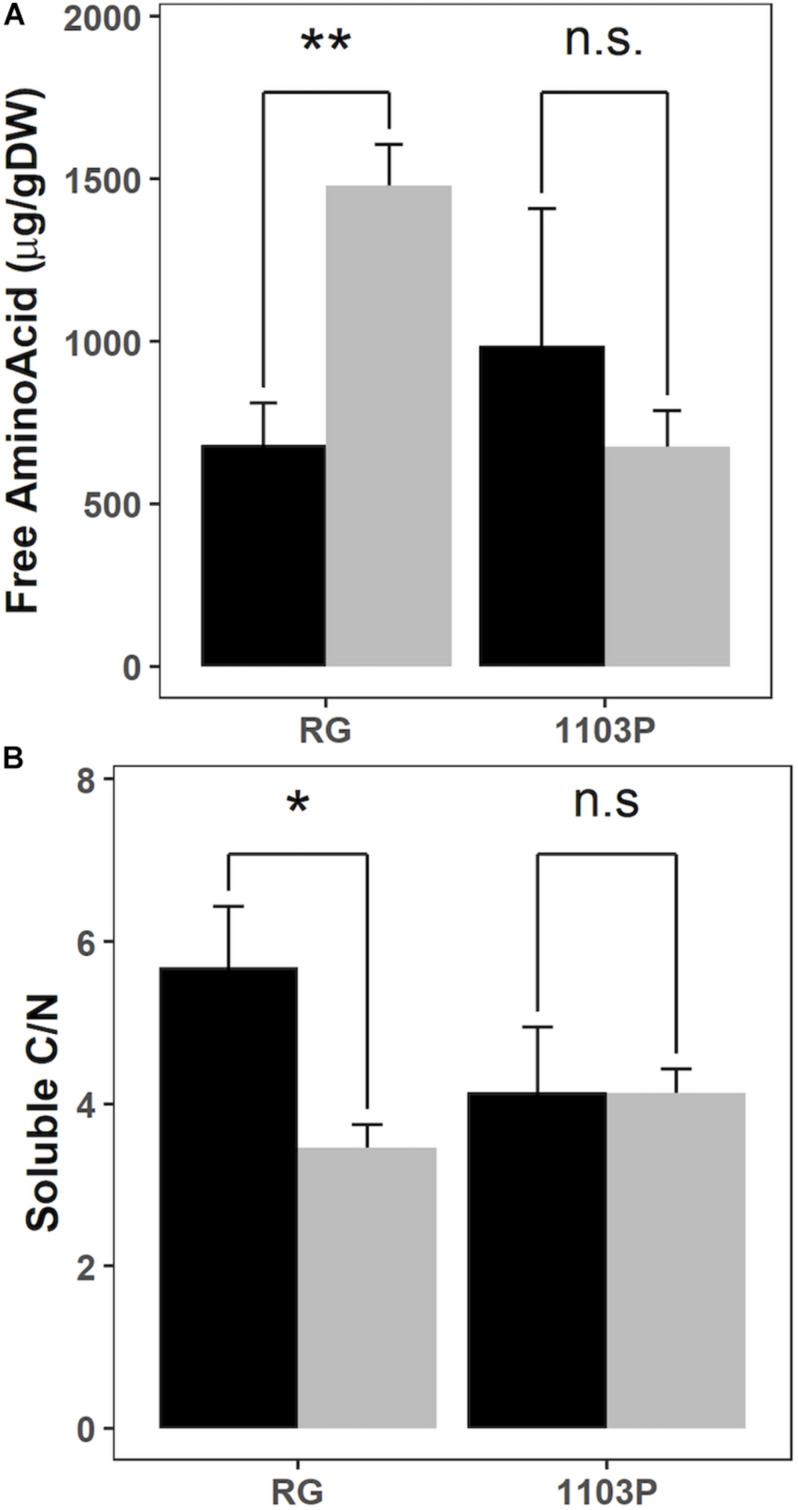
Evolution of free amino acids concentration **(A)** and soluble C/N calculated as TSC/(FAA + NO3) **(B)** in root tips of RG and 1103P before (black) and 24 h after (gray) 1 mM of nitrate resupply (mean ± se; *n* = 4). Asterisks show significant difference between rootstocks at each time point (Welch paired test, **P < 0.01,*P < 0.05, n.s., non-significant).

Total Soluble Carbohydrate (TSC), starch, nitrate and Free Amino Acids (FAA) concentrations were compared in young roots before and after 24 h of nitrate resupply (Figure 4 and Supplementary Figure 2). The concentration of amino acids increased after nitrate induction only in RG but not in 1103P (Figure 4A). No other significant changes were observed between rootstocks or in response to nitrate resupply, but the TSC concentration was 35% higher in RG young roots. The ratio of soluble C/N, estimated by the formula TSC/(FAA + Nitrate), significantly decreased for RG after nitrate resupply whereas it stayed stable for 1103P (Figure 4B).

### ^15^N Root-to-Shoot Transport

**FIGURE 5:**
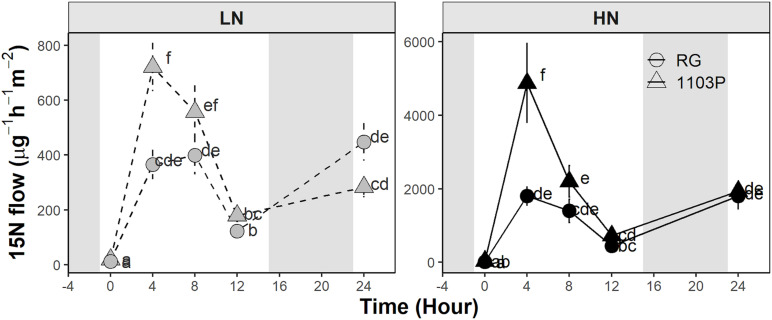
Flow of ^15^N measured in leaf xylem sap of Pinot Noir grafted to RG (circle) and 1103P (triangle) after 12 weeks of growth in LN (0.8 mM, gray) and HN (2.4 mM, black) solutions (mean ± se, *n* = 5–7). Letters refers to a significant difference between groups for each treatment separately (ANOVA, *P* < 0.05).

^15^N root-to-shoot transport was measured after 12 weeks of growth in grafted Pinot noir plants on RG and 1103P roots supplied with two levels of nitrate (Figure 5). Overall, the ^15^N flow rate followed the plant transpiration circadian rhythm and spiked after 4 h of labeling (5 h after sunrise) when the leaf transpiration was maximal (Supplementary Figure 3). At this time point, 1103P clearly showed a higher ^15^N transport than RG at both low and high nitrate availability (Figure 5). The leaf transpiration rate and ^15^N concentration in the xylem sap appeared to differ by rootstock at 4 h (Supplementary Figure 3). Rootstock affected carbon assimilation and stomatal behavior of the Pinot noir scion depending on nitrate availability (Supplementary Figure 3). In LN treatment, 1103P induced a higher carbon assimilation at 8 h and higher stomatal conductance at 0 and 8 h compared to RG. In HN treatment, stomatal conductance of 1103P was higher than that of RG at 0 and 4 h, while no differences were found for carbon assimilation (Supplementary Figure 3).

### Gene Expression in Root Under Steady-State Nitrate Supply

**FIGURE 6:**
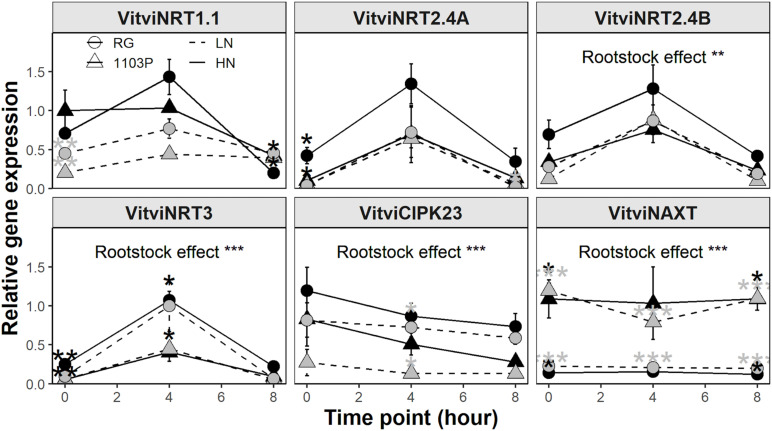
Relative gene expression of *VitviNRT1.1, VitviNRT2.4A, VitviNRT2.4B, VitviNRT3, VitviCIPK23*, and *VitviNAXT* in roots tips (mean ± se; *n* = 3) of RG (circle) and 1103P (triangle) after 12 weeks of growth in LN (0.8 mM, gray) and HN (2.4 mM, black) solutions. Time point correspond to collection time after exposure to labeled solutions (^15^N, 50%). Asterisks show significant difference between rootstocks at each time point for each nutrient solution (*t*-test, *P < 0.05, **P < 0.01; ***P < 0.001). Overall rootstock differences are presented (ANOVA, **P < 0.01; ***P < 0.001).

Except for *VitviNAXT*, all other genes examined showed a diurnal variation in the first 8 h of light (Figure 6 and Supplementary Figure 4). When considering all data together, *VitviCIPK23* and *VitviHY5* expression decreased, *VitviBTB* and *VitviNRT1.5* expression increased, whereas nitrate uptake and assimilation genes peaked at 4 h before decreasing at 8 h to a level similar to 0 h. *VitviNTR1.1*, *VitviNRT2.4A*, *VitviNRT2.4B*, *VitviHY5* showed differences between rootstock depending on nitrate availability. *VitviNRT1.1* expression was higher for RG only in LN treatment whereas *VitviNRT2.4A* and *VitviNRT2.4B* expressions were higher in RG only in HN treatment. A trend for more expression of *VitviHY5* at 0 h for 1103P was observed but it did not reach statistical significance (Supplementary Figure 4). For *VitviGS2*, *VitviNRT3*, *VitviNAXT*, and *VitviCIPK23*, the rootstocks differences were not dependent on nitrate availability. *VitviGS2*, *VitviNRT3*, and *VitviCIPK23* was higher for RG than 1103P whereas the opposite was observed for *VitviNAXT* expression. No differences between rootstocks were observed for *VitviBTB*, *VitviNR*, and *VitviNRT1.5* (Supplementary Figure 4).

### Biomass Allocation

**FIGURE 7:**
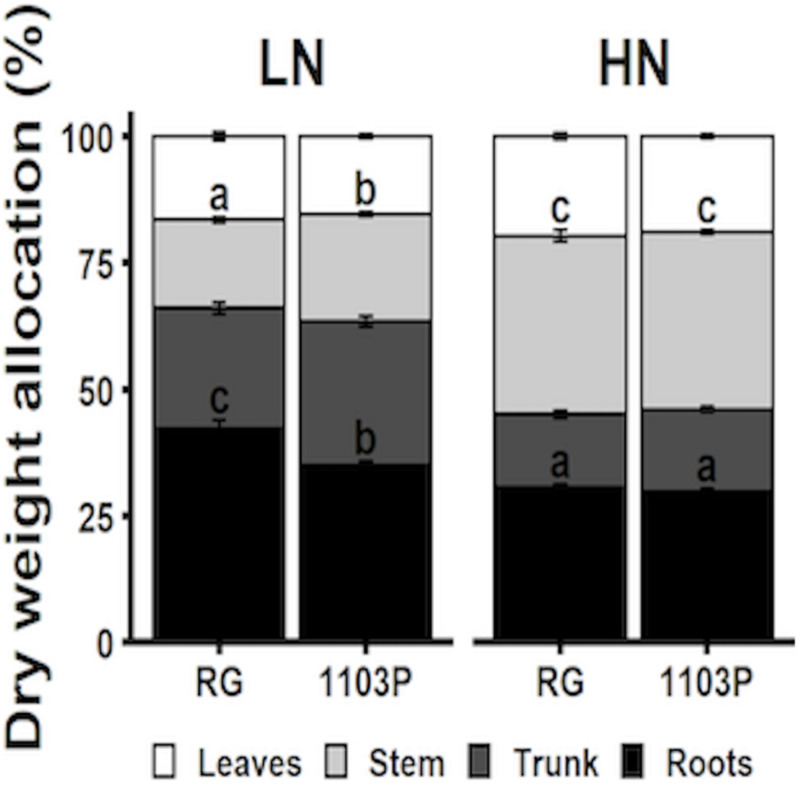
Allocation of total biomass (%) distributed in roots (dark), trunk (dark gray), stem (light gray), and leaves (white) of Pinot Noir grafted to RG and 1103P after 12 weeks of growth under LN (0.8 mM, gray) and HN (2.4 mM, black) solutions (*n* = 6–8, mean ± se). Letters when present shows significant interaction response between rootstocks and treatment per tissue (two-way ANOVA, *P* < 0.05). Absolute dry biomass allocation is provided in Supplementary Figure 6.

Biomass allocation to roots, trunks, stems and leaves were measured for Pinot noir grafted to RG and 1103P after 12 weeks of growth under LN and HN treatments (Figure 7). Nitrate treatment affected biomass allocation in all tissues in a similar manner between rootstocks. Regardless of the rootstock, allocation of biomass was greater in roots and trunks and lesser in stems and leaves under N-limitation (LN) in comparison with HN treatment. We also observed a greater allocation of biomass to root tissues in RG at the expense of stem and trunk tissues. Lower allocation of biomass to the stem in RG under LN treatment is supported by the weekly measure of stem length during the growth period, where significant difference between rootstocks appeared after the fifth week of LN treatment (Supplementary Figure 5). Total plant biomass increased in response to increasing nitrate availability but did not differ between rootstocks (Supplementary Figure 6).

### Metabolite Allocation

Nitrate, FAA, TSC, and starch concentrations were measured in leaves and roots after 12 weeks of growth under LN and HN treatment (Supplementary Figure 7). In leaves, N limitation (LN) enhanced nitrate and starch accumulation and decreased TSC and FAA concentration. In roots, only the concentration of FAA was affected by the N treatment. Increasing N availability, increased FAA concentration in roots, and the intensity of the response was higher for 1103P than RG (Table 3).

**TABLE 3:** Nitrate, FAA, TSC and starch as affected by Nitrogen treatment (HN and LN) and Rootstocks (1103P and RG) in leaves and root samples after 12 weeks of nitrogen supply.

Rootstock-Nitrogen Treatment	Nitrate (mg.g^–1^DW)	FAA (μg.mg^–1^DW)	TSC (mg.g^–1^DW)	Starch (mg.g^–1^DW)
**Leaves**				
1103P-LN	13.53 ± 0.66	1.48 ± 0.07	78 ± 2.6	339.2 ± 22.4
1103P-HN	9.14 ± 0.57	4.57 ± 0.45	103.5 ± 1.4	240.4 ± 12.9
Mean^*a*^	11.33	3.02	90.75	289.9
RG-LN	15.21 ± 0.91	1.99 ± 0.16	90.8 ± 2.2	279.1 ± 12.6
RG-HN	7.87 ± 0.31	5.71 ± 0.97	112.6 ± 1.8	217.3 ± 14.9
Mean^*a*^	11.54	3.85	101.7	248.2
**Roots**				
1103P-LN	8.39 ± 0.38	2.59 ± 0.24	40.1 ± 2.9	111.1 ± 8.1
1103P-HN	7.17 ± 0.44	6.77 ± 0.87	44.9 ± 2.5	108.9 ± 11.4
Mean^*a*^	7.78	4.68	42.5	110
RG-LN	7.23 ± 0.35	2.86 ± 0.24	51.1 ± 3.2	137.6 ± 12.0
RG-HN	7.26 ± 0.18	4.50 ± 0.53	47.8 ± 1.9	102.0 ± 7.8
Mean^*a*^	7.24	3.68	49.9	119.8
**Significance^*b,c*^**				
**Leaves**				
Rootstock	ns	ns	***	*
Treatment	***	***	***	***
Rootstock × Treatment	*	ns	ns	ns
**Roots**				
Rootstock	ns	*	*	ns
Treatment	ns	***	ns	ns
Rootstock × Treatment	ns	*	ns	ns

^*a*^Mean Across the treatment per rootstock. ^*b*^Significance of rootstock and treatment effects, and interactions (*p* > F; ns, not significant). ^*c*^Significance code: *<0.05, **<0.01, ***<0.001.

**FIGURE 8:**
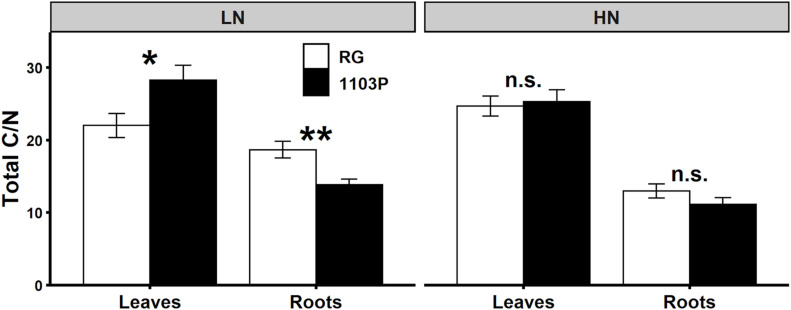
Ratio of total C/N in leaves and roots of Pinot Noir grafted to RG (white) and 1103P (black) after 12 weeks of growth under LN (0.8 mM) and HN (2.4 mM) solutions (*n* = 6–8, mean ± se). Total C is the addition of TSC and starch, and total N is the addition of FAA and nitrate. Asterisks show significant difference between rootstocks for each tissue and each treatment (*t*-test, *P < 0.05, **P < 0.01; n.s., non-significant).

Carbon allocation in leaves and roots was influenced by the rootstocks. For RG, TSC concentration in leaves and in roots was higher than 1103P whereas the opposite was observed for starch concentration in leaves. These differences in carbon metabolite preference and allocation between rootstocks seem accentuated in LN condition (Supplementary Figure 7). Total C/N ratio, estimated by the formula (TSC + Starch)/(FAA + Nitrate), illustrated the different rootstock strategy in allocation of C and N metabolites between leaves and roots (Figure 8). While total C/N ratio in leaves and roots were not dependent on the rootstock under HN, N limitation resulted in higher proportion of C metabolites in leaves for 1103P and in roots for RG.

## Discussion

The overall objective of our study was to compare nitrate uptake and its root-to-shoot transport between two rootstocks known to differ in their capacity to stimulate scion growth in grafted grapevines. Our study revealed that nitrate uptake capacity and biomass adaptation to N-limiting conditions are enhanced in the less vigorous rootstock (RG). These differences can be explained by a greater partitioning of C metabolites to the roots in RG as compared to 1103P. By contrast, the transpiration rate and N loading into xylem sap contributed to higher root-to-shoot transport of N in the vigorous rootstock (1103P). Higher transport of N to leaves with 1103P rootstock will promote N assimilation that can maintain shoot growth under LN conditions and increase FAA storage in roots under HN conditions. Overall, rootstocks did not affect the total plant biomass but appeared to contribute to a modification of biomass allocation in N-limited conditions. This is likely caused by a different ability of the rootstocks to control C and N metabolism and/or to transport water and mineral nutrients to the scion.

### Nitrate Uptake in Grapevine

Our experiments reinforce the established knowledge regarding induced nitrate uptake mechanisms in young grapevine roots. Previous studies demonstrated that grapevine HATS and LATS were induced after nitrate resupply (Yang et al., 2007; Pii et al., 2014; Tomasi et al., 2015; Kulmann et al., 2020), and their kinetic parameters were dependent on both rootstock and scion genotypes (Tomasi et al., 2015; Kulmann et al., 2020) or under spatial restricting conditions impeding the growth of the root system (Yang et al., 2007). Our results confirm the induction of both HATS and LATS and a faster response to the nitrate resupply compared to what is observed in other woody species (Tomasi et al., 2015).

Our work also revealed a dual Michaelis-Menten curve under low nitrate availability (<1 mM), which seems to validate that grapevine HATS consists of more than one high affinity nitrate transporter (Tomasi et al., 2015). In Arabidopsis *thaliana*, NRT2.1 is the major HATS controlling 75% of nitrate uptake under low nitrate availability (Cerezo et al., 2001; Filleur et al., 2001; Li et al., 2007). In response to heterogenous and homogenous N resupply, the expression of VitviNRT2.4A, of AtNRT2.1 putative ortholog, is higher in RG than in 1103P (Cochetel et al., 2017, 2019; Figure 3). Because the difference in nitrate uptake was observed only during the second phase of the HATS kinetic pattern (Figures 2C,D), it is possible that VitviNRT2.4A is responsible for HATS activity in this concentration range. Though, it is still unclear whether VitviNRT2.4A acts alone in the higher nitrate uptake of RG. *VitviNRT3*, the closest ortholog of *AtNRT3*, shows an identical induction pattern of expression to *VitviNRT2.4A* (Figure 3). AtNRT3 is essential to regulate the activity of HATS by formation of a tetrameric protein complex with AtNRT2.1 (Orsel et al., 2006; Yong et al., 2010; Kotur et al., 2012). Further experiments will be needed to determine the contribution of each transporter in the inducible HATS activity in young primary roots of grapevine.

Our results confirm the differential regulation of *VitviBTB* between rootstocks upon nitrate resupply (Figure 3) as proposed by Cochetel et al., 2017. However, neither nitrate availability nor rootstock genotype affected *VitviBTB* expression under steady state N supply (Supplementary Figure 4). Gene regulation of *AtBT2*, the closest homolog of *VitviBTB* in Arabidopsis thaliana, is dependent on the direct activity of NIN-LIKE PROTEIN (NLP) transcription factors (Sato et al., 2017), which modulates the majority of nitrate signaling and assimilation genes during the Primary Nitrate Response (PNR) (Marchive et al., 2013). AtBT2 was shown to be a negative regulator of Nitrogen Use Efficiency (NUE) decreasing the expression of both *AtNRT2.1* and *AtNRT2.4*, and the nitrate uptake under low nitrate availability (Araus et al., 2016). In fact, AtBT2 protein mediates multiple responses to nutrients, stresses and hormones (Mandadi et al., 2009) and may function as ubiquitin ligase to target specific proteins for degradation in response to calcium signaling (Misra et al., 2018) or UV-B treatment (An et al., 2019). Upregulation of *VitviBTB* only in RG could be the consequence of the more pronounced transcriptomic reprogramming generally observed during PNR among grapevine rootstocks (Cochetel et al., 2017). Further studies focusing at the protein level should be considered to determine the contribution of post-translational modifications to the different behavior of rootstocks to nitrate.

### Responsiveness of Riparia Gloire to N Availability Is Greater Than 1103P by Remobilizing C Metabolites Toward the Roots

The marked response of nitrate uptake and gene expression for nitrate transporters in RG roots (Figures 1–3) can be explained by differences in the management of C and N metabolites (Figure 4 and Supplementary Figure 2). We observed that FAA accumulated only in RG roots 24 h after nitrate induction (Figure 4A). The induced expression of *VitviGS2* ortholog in RG roots strengthens the hypothesis for enhanced N assimilation. N assimilation genes are known to be upregulated in roots by the level of nitrate and soluble sugars (Lejay et al., 2008; Bussell et al., 2013), and downregulated by amino acids (Tegeder and Masclaux-Daubresse, 2018). Overall, the greater soluble C/N ratio observed at t0h in RG (Figure 4B) might suggest a better or more rapid ability to respond to nitrate resupply than 1103P. This greater ratio was more associated with the TSC concentration that is 35% higher in RG roots compared to 1103P at t0h. Higher level of sugars in roots could enhance the nitrate uptake rate and gene regulation of nitrate transporters (Lejay et al., 1999; Guo et al., 2017), which could contribute to the differences in gene expression observed between the two rootstocks for *VitviNRT2.4A* and *VitviNRT3*.

A strong connection between total C/N ratio in root and plant biomass allocation is observed under steady state N supply. Under LN, higher TSC concentration (Supplementary Figure 7) is associated to higher biomass allocation toward the roots in RG rather than in 1103P. These differences in total C/N ratio disappear in HN availability consistent with similar biomass allocation for both rootstocks (Figure 7). Although biomass partitioning is known to respond to C/N ratio in grapevine (Grechi et al., 2007), this is the first report describing rootstock specificity in the allocation of C metabolites in roots without affecting the overall plant biomass. Root development in RG will compete with shoot growth for C and N resources especially in N-limited conditions with a greater tendency of RG to reduce the nitrate in roots. This could explain the lower shoot vigor typically conferred to the scion by RG, as opposed to poor phloem compatibility reducing C allocation to roots and reducing the overall size of the root system (Bieleski, 2000; Olmstead et al., 2006). However, more work is needed to confirm this, and whether or not such a mechanism only operates in young vines. Interestingly, the rate of photoassimilation or C content in leaves cannot explain the better root allocation of C in RG (Supplementary Figures 3, 7). However, rootstocks can actively modulate C flux through hormonal regulation. In grapevine, abscisic acid enhances C allocation toward roots and berries (Moreno et al., 2011), possibly by the regulation of sugar-related SWEET transporters (Meteier et al., 2019). Although, we did not validate this hypothesis, the lower stomatal conductance and transpiration rates under HN for RG during the highest transpiration period of the day (Supplementary Figure 3) suggests that a higher level of abscisic acid was present in the scion with RG, which in turn could promote the transport of sugars toward the roots as proposed by Meteier et al. (2019). A stomatal closure induced by ABA could reduce the rate of photoassimilation in leaves, which in turn would reduce the flow of carbon moving toward sinks like roots. More research is needed to understand rootstock effect on hydraulic properties and C and N source/sink strength between shoot and root growth.

### 1103P Rootstock Favors N Flux Toward Scion

Regardless of N availability, N flow in leaf xylem sap (Figure 5) was higher in 1103P, especially at 4 h when leaf transpiration is at the maximum (Supplementary Figure 3). Despite differences in N flow rate between rootstocks, nitrate and FAA were almost identical in scion leaves. This result could suggest that 1103P has a greater rate of assimilation of the newly transported nitrate that may promote shoot growth under limited conditions (Figure 7). Under non-limiting conditions, both rootstocks fulfilled the N demand of the scion in terms of N assimilation, but higher N flow of plants grafted to 1103P may translate into a greater reallocation of FAA in 1103P roots. Further investigations are needed to determine if the ability of 1103P to reallocate more FAA in the roots under these conditions is consistent across years. This adaptive strategy could be important for sustaining shoot growth in N-fluctuating field environments. Further, it is unknown whether this strategy leads to more developed root systems in 1103P which could explain its influence on shoot vigor; this needs to be validated in field experiments.

In the N-steady state experiment, differences in N xylem flow between rootstocks were noted through variations in N xylem sap concentration and the transpiration stream (Supplementary Figure 3). While xylem loading and transpiration are independent processes, their cumulative effects mutually impact N xylem flow (Macduff and Bakken, 2003; Siebrecht et al., 2003). We did not measure N uptake in this experiment, so nitrate uptake and xylem loading cannot be estimated. The greater expression of *VitviNRT3* orthologs, a possible activator of the HATS, suggests a greater N-uptake in RG in this steady-state experiment (Figure 6). However, the N concentration in the xylem sap was lower in RG, and the concentration of nitrate and FAA did not suggest a higher N accumulation in RG roots (Supplementary Figure 7). The greater nitrate uptake capacity measured from root tips (Figures 1, 2) may not translate into a greater overall nitrate uptake into the roots. Recent advances on NRT1.1 demonstrate that its phosphorylation status determines whether it will act as nitrate or auxin transporters. Phosphorylation of NRT1.1 caused by CIPK23 in Arabidopsis (Ho et al., 2009), promotes the basipetal transport of auxin in fine roots, thereby inhibiting the formation of lateral roots (Liu and Tsay, 2003; Bouguyon et al., 2015, 2016; Zhang et al., 2019). We observed that the expression of the AtCIPK23 ortholog, *VitviCIPK23* (Griesser et al., 2017; Xi et al., 2017), was higher in RG root tips regardless of N availability (Figure 6). While the N uptake in RG seems higher at the root tip level, this rootstock was shown to limit the formation of lateral roots when compared with 1103P (Cochetel et al., 2019), which could limit nitrate uptake, and thereby could reduce the global N uptake. Moreover, the phosphorylation of NRT1.1 by CIPK23 limits its uptake capacity when N is available (Wang C. et al., 2018), which could explain the lack of FAA accumulation in RG root under HN.

### Could VitviNAXT Be a Xylem Loading NO_3_ Transporter?

In our experiments, *VitviNAXT* transcript abundance was not affected by N resupply and N plant status whereas its expression was significantly higher in 1103P than in RG (Figures 3, 7). In Arabidopsis, NAXT1/NPF2.7 has been demonstrated to regulate nitrate efflux to the media (Segonzac et al., 2007). Function of *VitviNAXT* as a nitrate efflux transporter appears unlikely, as its expression is much higher in 1103P in which we observed higher N translocation rate. VitviNAXT has not been functionally characterized in grapevine, but it may participate in rootstock chloride exclusion of rootstocks under salt stress (Henderson et al., 2014). AtNAXT1 is a NRT1 transporter part of the NPF2a clade containing seven members (Segonzac et al., 2007). Three of them demonstrated ion transport activity. Two members, NPF2.4 (Li B. et al., 2016) and NPF2.5 (Li et al., 2017), have been characterized in Arabidopsis and participate to chloride loading into the xylem and chloride efflux into the media, respectively. The third member, AtNPF2.3, contributes more to the nitrate xylem loading under salt stress (Taochy et al., 2015). KO mutants of AtNRT1.5/NPF7.3 showed only partial limitation of translocation rate to the xylem (Lin et al., 2008); its loss of function under salt stress was found to be compensated by the functional redundancy of AtNPF2.3 (Taochy et al., 2015). Like AtNPF2.3, *VitviNAXT* is not responsive to salt stress (Henderson et al., 2014). However, functional characterization will be needed to determine whether *VitviNAXT* can transport nitrate.

Rootstock differences in N translocation to the shoot is partially explained by their impact on scion transpiration (Supplementary Figure 3). In the HN condition, 1103P displayed increased scion stomatal conductance and transpiration leading to higher xylem sap flow than RG, especially during high transpiration demand (at 4 and 8 h). In the LN condition, this observation was true only at 8 h and could be associated with partial stomatal closure for RG probably caused by lower leaf water potential at 4 h (Supplementary Figure 3). This transpiration behavior under LN conditions during a specific time of the day in 1103P were also confirmed when the daily transpiration rate of the plants was calculated 1 day before the ^15^N was supplied (Supplementary Figure 8). Altogether, these higher transpiration patterns observed with 1103P would facilitate greater root-to-shoot transport of nutrients and hormones but also increases the mass flow movement of soluble nutrients in the soil toward the root surface (Cramer et al., 2009). In addition to a deeper rooting phenotype for 1103P (Cochetel et al., 2019), the promotion of scion transpiration with 1103P might increase nutrient acquisition by facilitating bulk flow of soluble nutrients like nitrate in soil toward the root surface. In grapevine, scion transpiration and shoot vigor are tightly correlated to the root hydraulic conductance and the activity of root aquaporins (Vandeleur et al., 2009; Gambetta et al., 2012; Perrone et al., 2012; Zhang et al., 2016). Nitrate availability modulates transcription and/or activity of root aquaporins in many species to optimize nitrate uptake in homogenous or heterogenous availability (Gloser et al., 2007; Gorska et al., 2008a,b; Ishikawa-Sakurai et al., 2014; Li G. et al., 2016). Thus, the variation of root hydraulic conductivity under the control of aquaporin activity among rootstocks (Lovisolo et al., 2008; Gambetta et al., 2012) may also contribute to the rootstock performance in its capacity to facilitate the transport of nitrate toward the upper part of the plants in limited and heterogenous nitrate availability.

## Conclusion

The physiological communication between scions and rootstocks is essential to regulate plant development and the acquisition of C and N. In our experiments, the contribution of the rootstock to scion vigor was observed only when N supply was limited and resulted in the alteration of biomass allocation rather than total biomass production. Biomass allocation to the roots and remobilization of C resources from shoot-to-root are part of the adaptive strategy under N-limiting conditions to stimulate N uptake in plants. Here, we demonstrated that two commonly used commercial rootstocks possess distinct strategies to transport, assimilate, and allocate N in the plant. Our findings also suggest that the greater capacity of 1103P to translocate N to shoots, possibly through an increase of transpiration, would allow greater shoot growth under limiting conditions. The capacity of different rootstocks to affect the regulation of transpiration to the scion may be considered a major contributing factor to explain the differences observed in grapevine C and N status. Further experiments including manipulation of grapevine water status should be conducted to validate the influence of transpiration on N uptake, its root-to-shoot transport, and its assimilation in grapevines. By contrast, the higher responsiveness of RG with its capacity to put more N resources in roots rather than in shoots appears to be a more conservative strategy to allocate N to perennial tissues. In both rootstocks, it is unclear whether these mechanisms are controlled directly by the rootstock itself or by interactions with the scion through a feedback loop mechanism. Further investigation is needed to clarify the roles played by both scions and rootstocks on the regulatory activity of the other partner in grafted grapevines with respect to C and N acquisition. Ultimately, whether these distinct behaviors between both rootstocks are maintained over seasons and in the field have yet to be addressed.

## Data Availability Statement

The original contributions presented in the study are included in the article/Supplementary Material, further inquiries can be directed to the corresponding author.

## Author Contributions

LR, RS, PS, and LD conceived and planned the study. LR, RS, and LD collected the samples and LR processed the samples for nitrate uptake, gene expression, biomass allocation, and metabolite and physiological measurements. LR performed all the statistical analyses and wrote the body of the manuscript with LD. All authors reviewed and approved the manuscript.

## Conflict of Interest

The authors declare that the research was conducted in the absence of any commercial or financial relationships that could be construed as a potential conflict of interest.

## Abbreviations

1103P: 1103 Paulsen
RG: Riparia Gloire
LATS: Low Affinity Transporter System
HATS: High Affinity Transporter System
FAA: Free Amino Acids
TSC: Total Soluble Carbohydrates
ns: not significant
se: standard error.
