# Supplementary material for: Nitrate Uptake and Transport Properties of Two Grapevine Rootstocks With Varying Vigor
Source: Front Plant Sci. 2021 Jan 18;11:608813. doi: 10.3389/fpls.2020.608813 (PMC7847936; doi:10.3389/fpls.2020.608813)
Supplement: Supplementary file 1 [file Data_Sheet_1.docx]

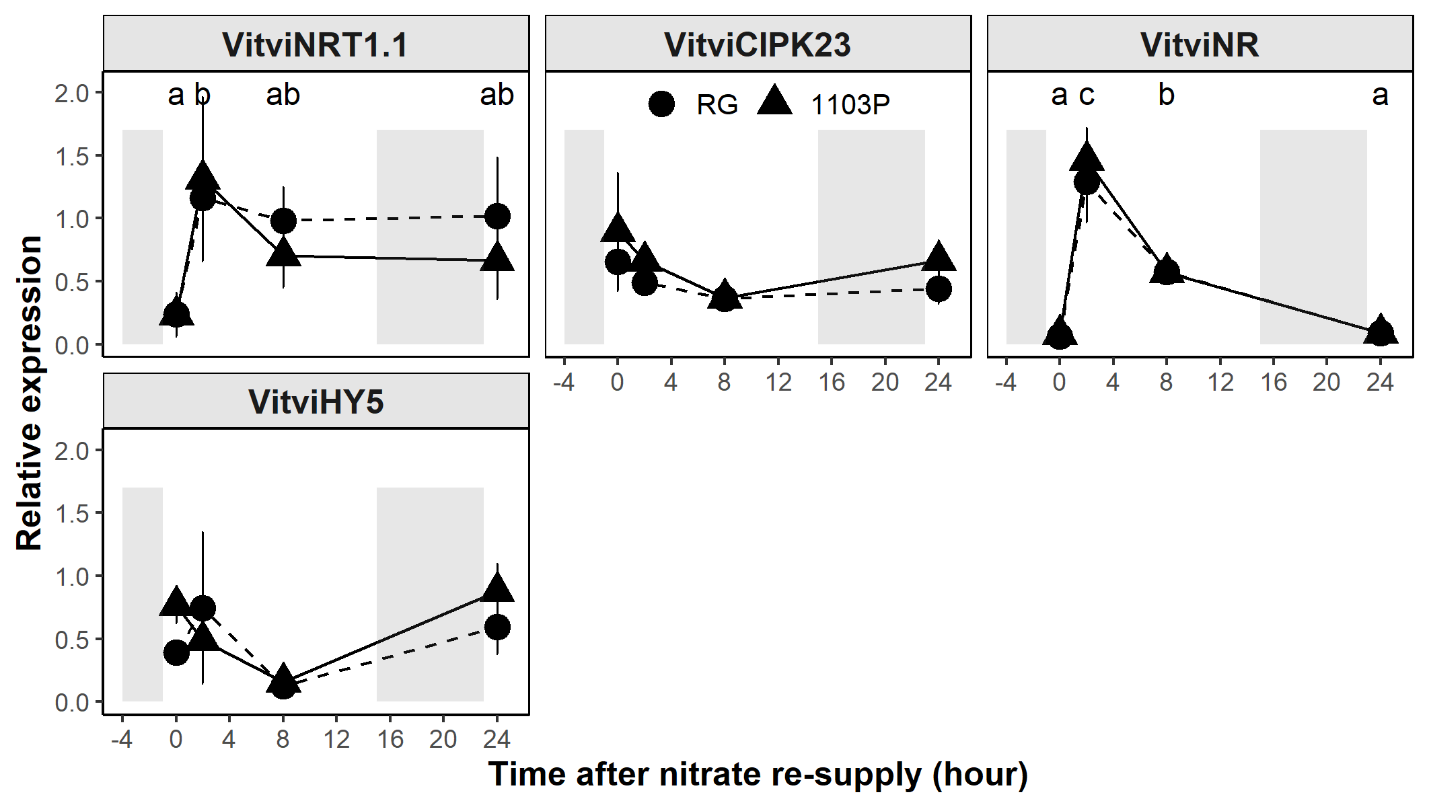


Supplementary Figure 1. Relative gene expression of *VitviNRT1.1, VitviCIPK23, VitviNR* *VitviHY5* in roots tips (mean ± se; n=3) of RG (circle) and 1103P (triangle) in response to nitrate resupply. Indicated time correspond to exposure duration to 0.5mM Ca(NO_3_)_2_ after a 10 days period of N-starvation; shaded areas represent dark period. Letters refers to a significant difference between time point considering the two rootstocks (ANOVA, P < 0.05).


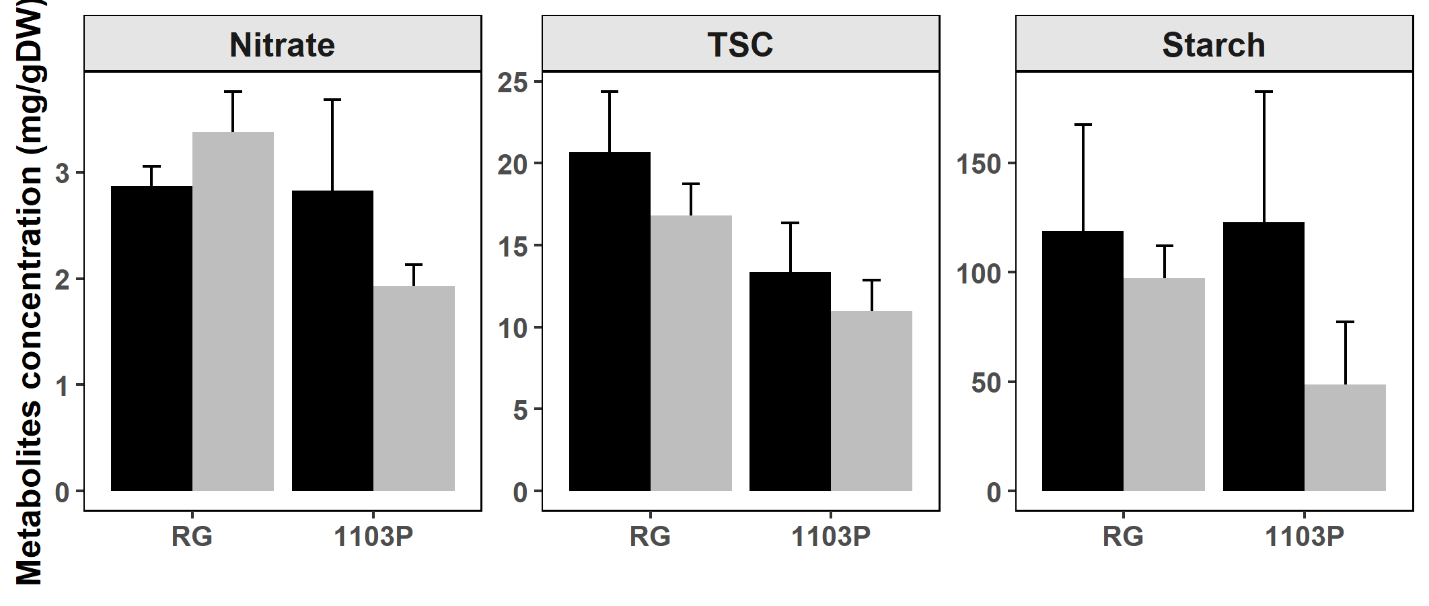


Supplementary Figure 2. Evolution of nitrate, TSC, starch in root tips of RG and 1103P before (black) and 24 hours after (grey) 1mM of nitrate resupply (mean ± se; n=4).


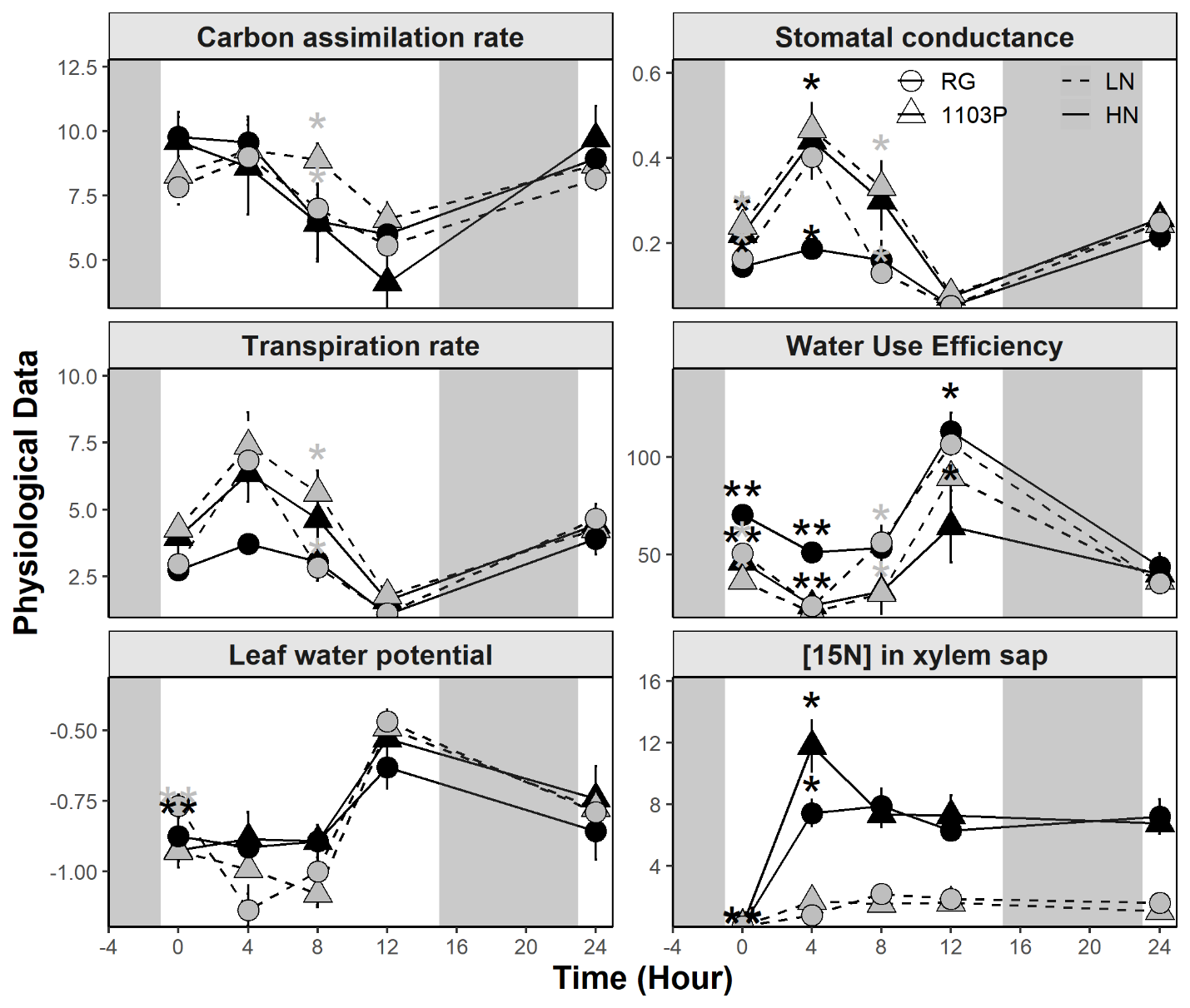
Supplementary Figure 3. Physiological measurement of Pinot Noir grafted to RG (circle) and 1103P (triangle) after 12 weeks of growth in LN (0.8 mM, grey) and HN (2.4 mM, black) solutions (mean ± se, n = 5-7). Shaded areas represent dark period. Asterisks show significant difference between rootstocks at each time point for each nutrient solution (t-test, *: P < 0.05, **: P < 0.01). Units: Carbon assimilation rate (µmol.m^-2^.s^‑1^); Stomatal conductance (mol.m^‑2^.s^‑1^); Transpiration rate (mol.m^‑2^.s^‑1^); Water Use Efficiency (µmol_CO2_.mol^-1^_H20_); Leaf water potential (MPa); Concentration of 15N (µg.mL^-1^).

**
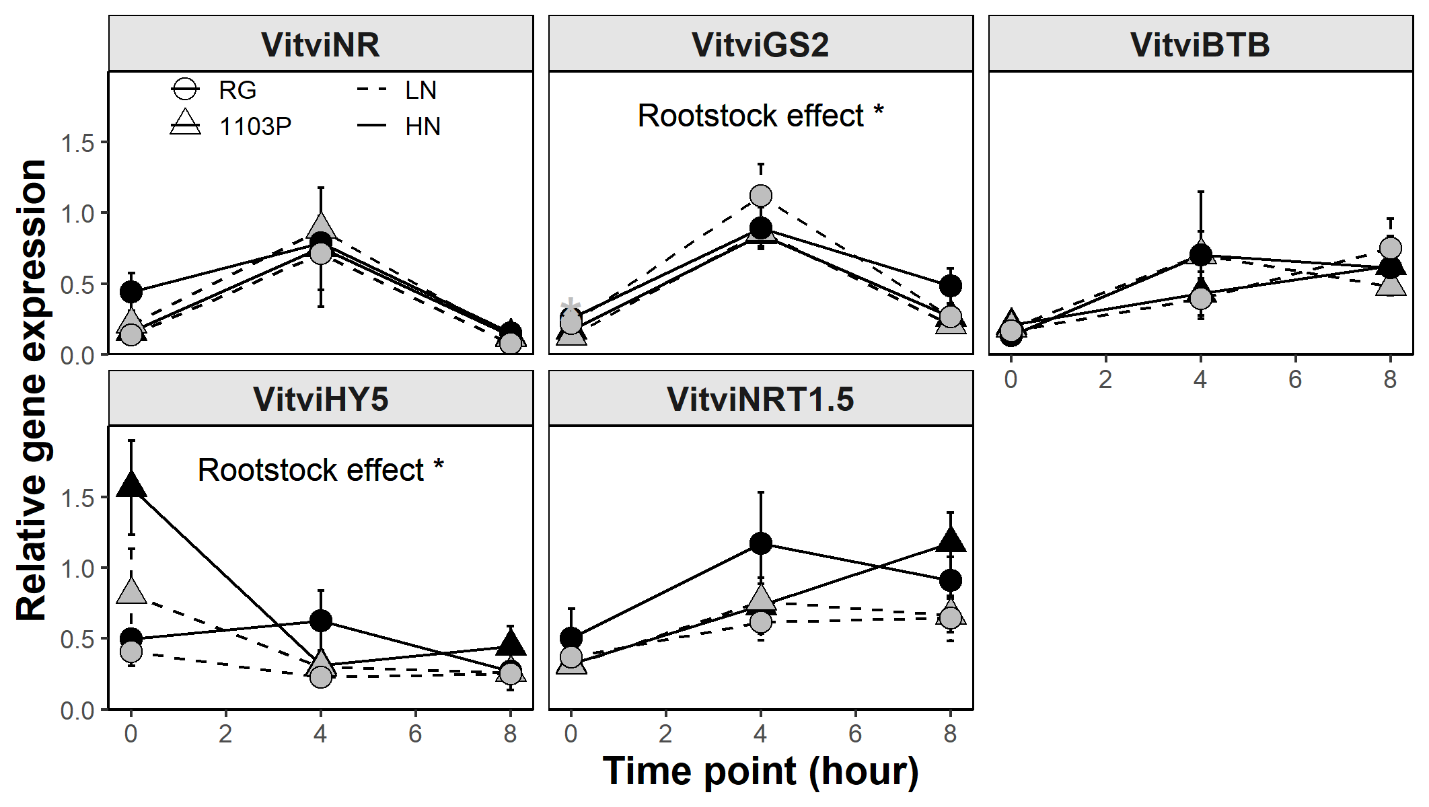
**Supplementary Figure 4. Relative gene expression of *VitviNR, VitviGS2, VitviBTB, VitviHY5* and *VitviNRT1.5* in roots tips (mean ± se; n=3) of RG (circle) and 1103P (triangle) after 12 weeks of growth in LN (0.8 mM, grey) and HN (2.4 mM, black) solutions. Time point correspond to collection time after exposure to labelled solutions (15N, 50%). Overall rootstock differences are presented (ANOVA, *: P < 0.05).


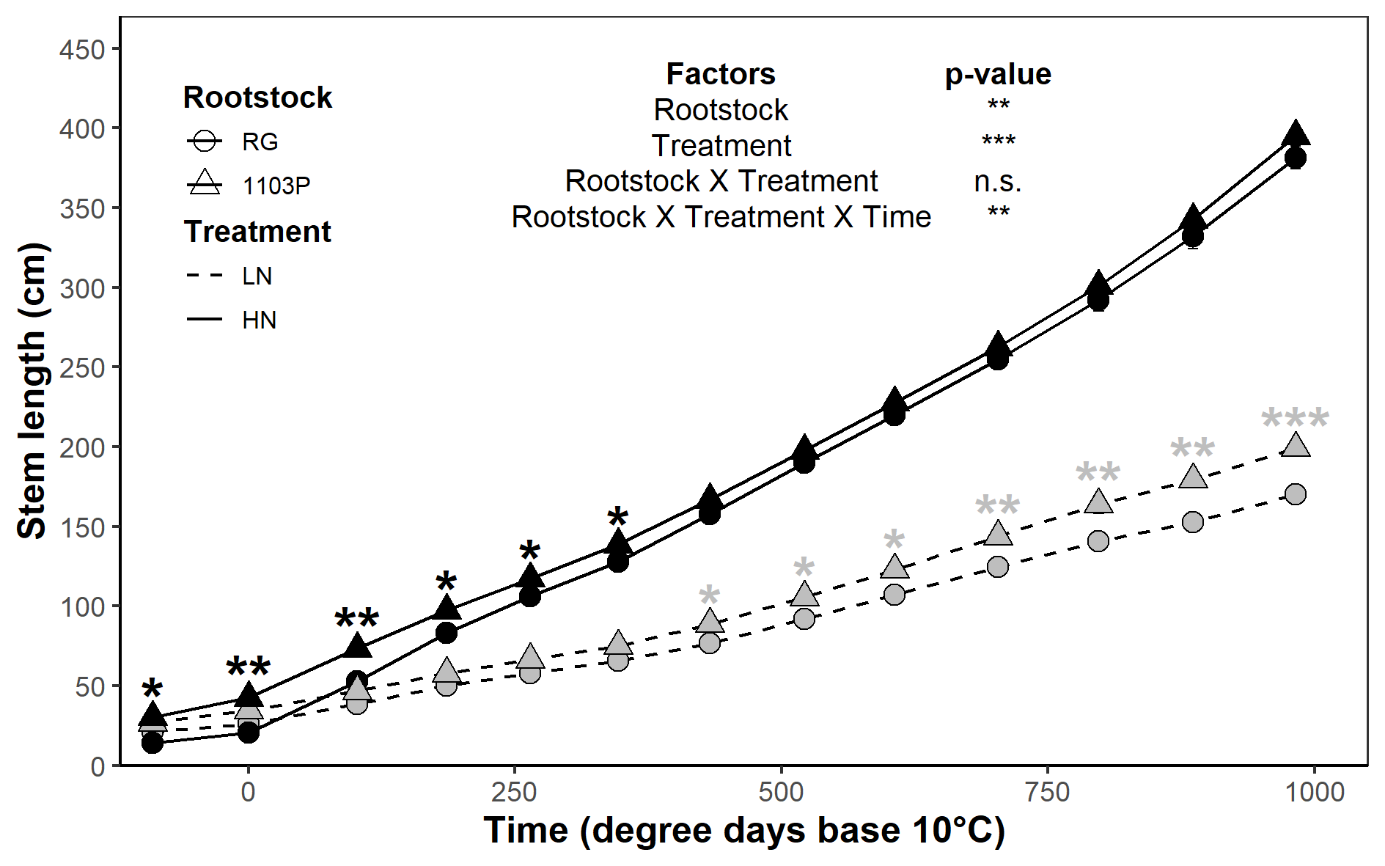
 Supplementary Figure 5. Stem length measurement of Pinot Noir grafted to RG (circle) and 1103P (triangle) during 11 weeks of growth in LN (0.8 mM, grey, dotted line) and HN (2.4 mM, black, line) solutions (mean ± se, n = 6-7). Duration of the experiment is expressed in degree days starting at the first treatment application. Results of a repeated ANOVA are presented, and asterisks show significant difference between rootstocks at each time point for each nutrient solution (t-test). *: P < 0.05, **: P < 0.01; ***: P < 0.001; n.s.: non-significant).


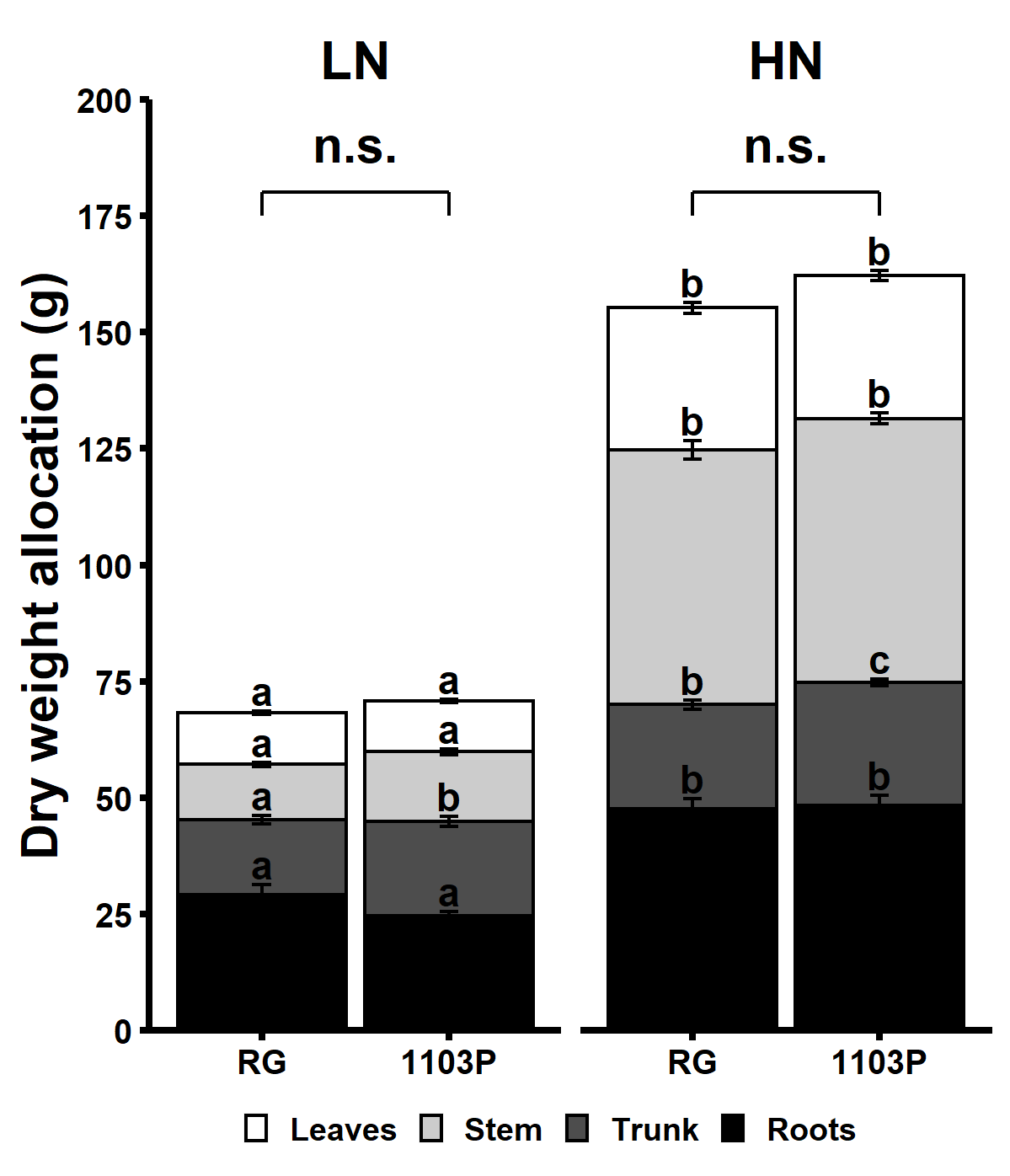


Supplementary Figure 6. Dry weight Biomass allocation (g) of roots (dark), trunk (dark grey), stem (ligth grey), and leaves (white) of Pinot Noir grafted to RG and 1103P after 12 weeks of growth under LN (0.8 mM) and HN (2.4 mM) solutions (n = 6-7, mean ± se). Letters shows interaction response between rootstocks and treatment per tissu (two-way ANOVA, P < 0.05). Total dry weight was compared between rootstocks for each treatment separatly (t-test; n.s.: non significant).


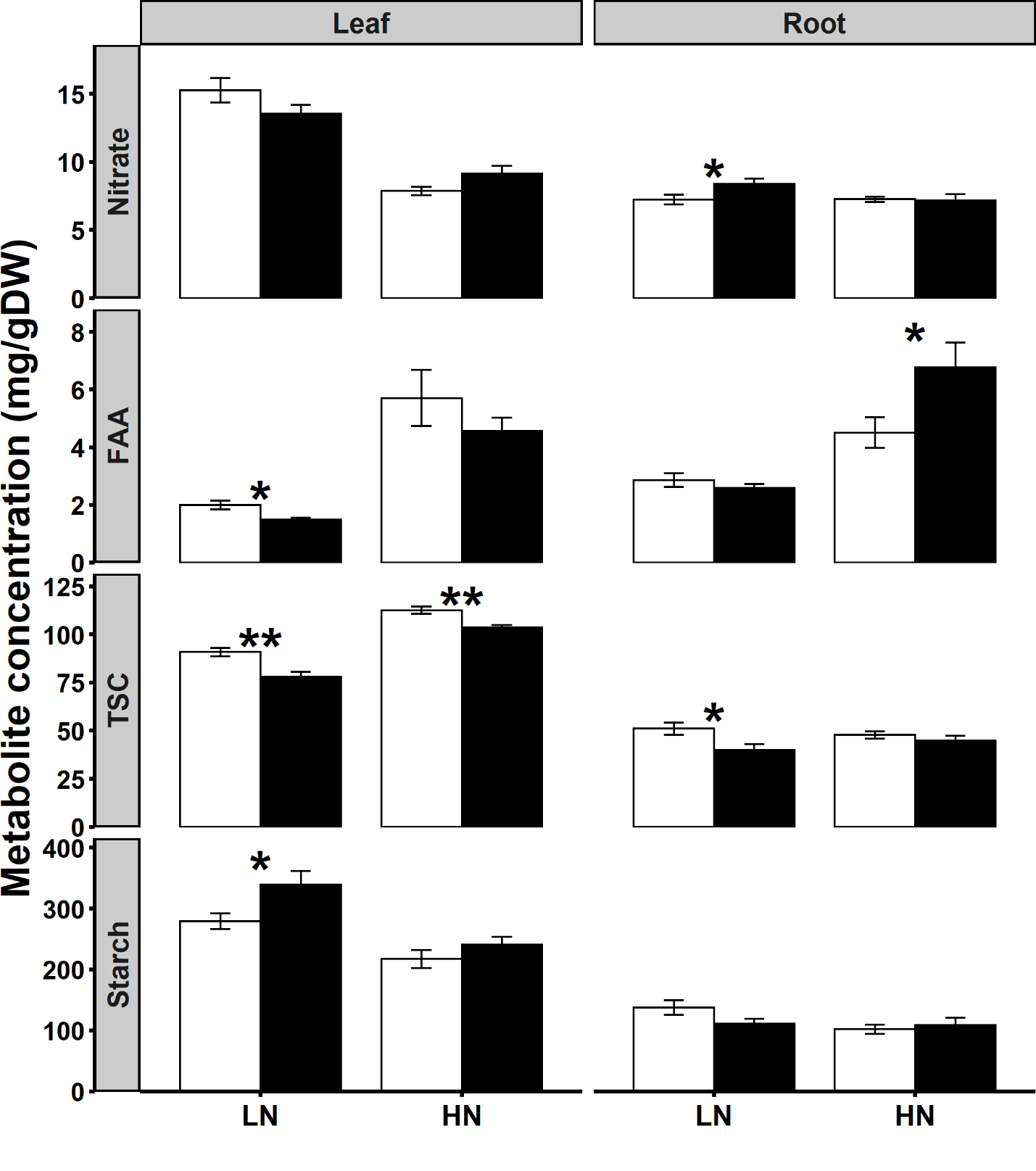


Supplementary Figure 7. Concentration of nitrate, FAA, TSC and starch in leaves and roots of Pinot Noir grafted to RG (white) and 1103P (black) after 12 weeks of growth under LN (0.8 mM) and HN (2.4 mM) solutions (n = 6-7, mean ± se). Asterisks show significant difference between rootstocks for each tissue and each treatment (t-test, *: P < 0.05, **: P < 0.01).


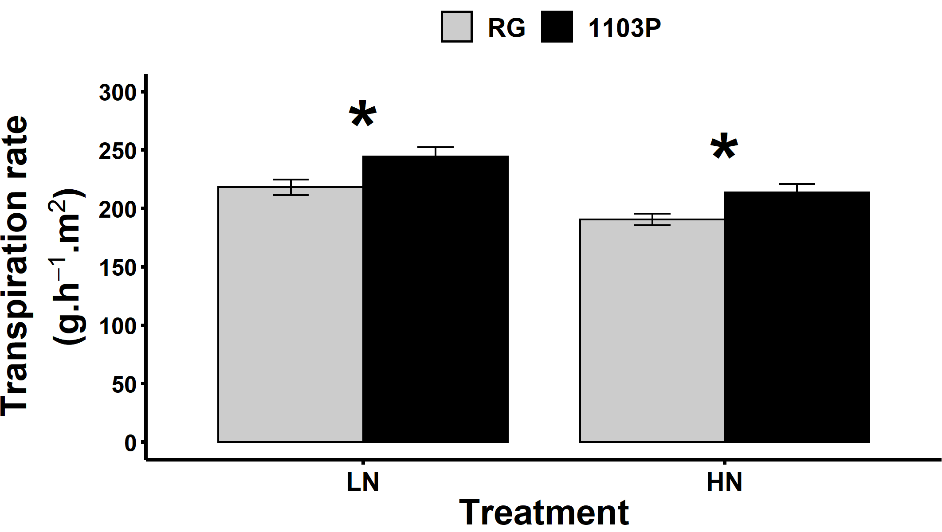


Supplementary Figure 8. Transpiration rate measured by weigh difference between 8:30 am and 6:00pm on Pinot Noir grafted to RG (grey) and 1103P (black) after 12 weeks of growth under LN (0.8 mM) and HN (2.4 mM) solutions (n = 6-7, mean ± se). Asterisks show significant difference between rootstocks for each treatment (t-test, *: P < 0.05).
